# In vitro antimicrobial susceptibility of clinical respiratory isolates to ceftazidime-avibactam and comparators (2016–2018)

**DOI:** 10.1186/s12879-021-06153-0

**Published:** 2021-06-23

**Authors:** D. Piérard, G. G. Stone

**Affiliations:** 1 Department of Microbiology and Infection Control, Universitair Ziekenhuis Brussel (UZ Brussel), Vrije Universiteit Brussel (VUB), Laarbeeklaan, 101, B-1090 Brussels, Belgium; 2 Pfizer Inc., Hospital Business Unit, Global Product Development, Groton Laboratories, 558 Eastern Point Rd, Groton, CT 06340 USA

**Keywords:** Ceftazidime-avibactam, *Enterobacterales*, *Pseudomonas aeruginosa*, Respiratory isolates, Antimicrobial susceptibility, Antimicrobial surveillance, Carbapenemase, Metallo-β-lactamase, Multidrug-resistant

## Abstract

**Background:**

This antimicrobial surveillance study reports in vitro antimicrobial activity and susceptibility data for a panel of agents against respiratory isolates of *Enterobacterales* and *Pseudomonas aeruginosa*.

**Methods:**

Isolates from respiratory specimens were collected in Africa/Middle East, Asia/South Pacific, Europe and Latin America between 2016 and 2018, as part of the Antimicrobial Testing Leadership and Surveillance (ATLAS) program. Broth microdilution methodology was used to quantify minimum inhibitory concentrations, from which rates of susceptibility were determined using EUCAST breakpoints (version 10). Rates of subsets with genes encoding β-lactamases (extended-spectrum β-lactamases [ESBLs], serine carbapenemases and metallo-β-lactamases [MBLs]) were also determined, as well as rates of multidrug-resistant (MDR) *P. aeruginosa*.

**Results:**

Among all respiratory *Enterobacterales* isolates, susceptibility to ceftazidime-avibactam, meropenem, colistin and amikacin was ≥94.4% in each region. For *Enterobacterales* isolates that were ESBL-positive or carbapenemase-positive/MBL-negative, ceftazidime-avibactam susceptibility was 93.6 and 98.9%, respectively. Fewer than 42.7% of MBL-positive *Enterobacterales* isolates were susceptible to any agents, except colistin (89.0% susceptible). Tigecycline susceptibility was ≥90.0% among *Citrobacter koseri* and *Escherichia coli* isolates, including all β-lactamase-positive subsets. ESBL-positive *Enterobacterales* were more commonly identified in each region than isolates that were ESBL/carbapenemase-positive; carbapenemase-positive/MBL-negative; or MBL-positive. Among all respiratory *P. aeruginosa* isolates, the combined susceptibility rates (susceptible at standard dosing regimen plus susceptible at increased exposure) were highest to ceftazidime-avibactam, colistin and amikacin (≥82.4% in each region). Susceptibility to colistin was ≥98.1% for all β-lactamase-positive subsets of *P. aeruginosa*. The lowest rates of antimicrobial susceptibility were observed among MBL-positive isolates of *P. aeruginosa* (≤56.6%), with the exception of colistin (100% susceptible). MDR *P. aeruginosa* were most frequently identified in each region (18.7–28.7%), compared with the subsets of ESBL-positive; carbapenemase-positive/MBL-negative; or MBL-positive isolates.

**Conclusions:**

Rates of susceptibility among the collections of respiratory *Enterobacterales* and *P. aeruginosa* isolates were highest to ceftazidime-avibactam, colistin and amikacin in each region. Tigecycline was active against all subsets of *C. koseri* and *E. coli*, and colistin was active against all subsets of *P. aeruginosa*. The findings of this study indicate the need for continued antimicrobial surveillance among respiratory Gram-negative pathogens, in particular those with genes encoding MBLs.

**Supplementary Information:**

The online version contains supplementary material available at 10.1186/s12879-021-06153-0.

## Introduction

Antimicrobial resistance among Gram-negative bacteria is a long-standing problem that needs to be monitored in an effort to preserve the efficacy of current antimicrobial agents. *Pseudomonas aeruginosa* and species of *Enterobacterales* are common causative pathogens of respiratory infections such as hospital-acquired bacterial pneumonia (HABP) and ventilator-associated bacterial pneumonia (VABP), and respiratory infections caused by antimicrobial-resistant *Enterobacterales* and *P. aeruginosa* are associated with higher patient mortality [1, 2]. Among isolates of *Enterobacterales* and *P. aeruginosa*, antimicrobial resistance can be mediated by the production of β-lactamases, for example, extended-spectrum β-lactamases (ESBLs), serine carbapenemases and metallo-β-lactamases (MBLs) [3].

Ceftazidime-avibactam is a combination antimicrobial agent comprising ceftazidime, a third-generation cephalosporin, and avibactam, a non-β-lactam β-lactamase-inhibitor. Avibactam inhibits Ambler class A, class C, and certain class D OXA-type β-lactamases, but not MBLs. Therefore ceftazidime-avibactam is active against ESBL- and serine carbapenemase-positive Gram-negative isolates, but not against MBL-positive isolates [4–6]. Ceftazidime-avibactam has been approved for the treatment of HABP and VABP, as well as complicated intra-abdominal infection (in combination with metronidazole) and complicated urinary tract infection (including pyelonephritis) [7, 8]. In Europe, ceftazidime-avibactam is also indicated for the treatment of infections due to aerobic Gram-negative organisms in adult patients with limited treatment options [8].

The aim of this study is to report in vitro antimicrobial activity and susceptibility data for a panel of antimicrobial agents against isolates of *Enterobacterales* and *P. aeruginosa* collected from respiratory specimens as part of the Antimicrobial Testing Leadership and Surveillance (ATLAS) program (2016–2018). Data on rates of resistant isolates will also be presented. The geographical regions of collection included in the analysis are Africa/Middle East, Asia/South Pacific, Europe and Latin America.

## Methods

Isolates from respiratory specimens were collected from hospitalised patients from participating study centers between 2016 and 2018 in four geographical regions (Africa/Middle East, Asia/South Pacific, Europe and Latin America). Only non-duplicate isolates of the organism considered to be the potential causative pathogen of the infection were included in the study. Demographic information recorded for each isolate included specimen source, patient age and sex, and type of hospital setting.

Isolates were collected and identified at the participating center, and pure cultures were shipped to the central laboratory (International Health Management Associates [IHMA] Inc., Schaumburg, IL, USA). The central laboratory then re-identified and confirmed bacterial species using matrix-assisted laser desorption/ionization time-of-flight mass spectrometry (Bruker Biotyper; Bruker Daltonics, Billerica, MA, USA), and performed antimicrobial susceptibility testing using self-manufactured frozen broth microdilution panels [9]. Ceftazidime-avibactam was tested with a fixed concentration of 4 mg/L avibactam with doubling dilutions of ceftazidime. All minimum inhibitory concentrations (MICs) were interpreted using version 10.0 of the European Committee on Antimicrobial Susceptibility Testing (EUCAST) clinical breakpoint tables [10]. Among the *Enterobacterales*, isolates of *Morganella* spp., *Proteus* spp., *Providencia* spp. or *Serratia* spp. were excluded from the analysis for colistin activity, due to intrinsic resistance. All isolates of *Enterobacterales* were included in the analysis for tigecycline activity, but tigecycline EUCAST breakpoints are only available for isolates of *Citrobacter koseri* and *Escherichia coli*. For isolates of *P. aeruginosa*, EUCAST have revised the susceptible breakpoints at the standard dosing regimen (S) for piperacillin-tazobactam, aztreonam, ceftazidime, cefepime, imipenem and levofloxacin [10], therefore isolates tested against these antimicrobial agents are categorized as either susceptible at increased exposure (I), or as resistant (R). For all antimicrobial agents tested against *P. aeruginosa*, the combined susceptibility rates (susceptible at standard dosing regimen plus susceptible at increased exposure) are presented here.

A multidrug-resistant (MDR) phenotype among isolates of *P. aeruginosa* was defined as resistance to one or more antimicrobial agent (given in parentheses) from three or more of the following antimicrobial classes: aminoglycosides (amikacin), carbapenems (imipenem or meropenem), cephalosporins (ceftazidime or cefepime), fluoroquinolones (levofloxacin) and β-lactam/β-lactamase inhibitor combinations (piperacillin-tazobactam).

*Enterobacterales* isolates with MIC values of ≥2 mg/L to meropenem were screened for genes encoding clinically-relevant β-lactamases (ESBLs: SHV, TEM, CTX-M, VEB, PER and GES; plasmid-mediated AmpC β-lactamases: ACC, ACT, CMY, DHA, FOX, MIR and MOX; serine carbapenemases: GES, KPC and OXA-48-like; and MBLs: NDM, IMP, VIM, SPM and GIM) using published multiplex PCR assays [11]. Additionally, isolates of *E. coli*, *Klebsiella pneumoniae*, *Klebsiella oxytoca* and *Proteus mirabilis* with MIC values of ≥2 mg/L to ceftazidime or aztreonam were screened for the same genes. *P. aeruginosa* isolates with meropenem MIC values of ≥4 mg/L were screened for genes encoding MBLs (IMP, VIM, NDM, GIM and SPM) and serine carbapenemases (KPC and GES) using published multiplex PCR assays [12]. All detected carbapenemase genes were amplified using flanking primers and sequenced, and sequences were compared against publicly available databases.

The following subsets of resistant isolates are presented: ESBL-positive *Enterobacterales*; ESBL-positive and carbapenemase-positive *Enterobacterales* (hereafter described as ESBL/carbapenemase-positive); carbapenemase-positive and MBL-negative *Enterobacterales* or *P. aeruginosa*; carbapenemase-positive and MBL-positive *Enterobacterales* or *P. aeruginosa* (hereafter described as MBL-positive) and MDR *P. aeruginosa*.

## Results

### Isolates collected

A total of 15,460 isolates from respiratory specimens (10,128 *Enterobacterales* and 5332 *P. aeruginosa*) were collected from a total of 51 countries in four geographical regions between 2016 and 2018. The number of centers in each participating country and the number of isolates collected by each center are presented in **Supplementary Table** **1**. More than half of isolates were collected in Europe (58.6%; *n* = 9055), followed by Asia/South Pacific (19.7%; *n* = 3040); Latin America (13.3%; *n* = 2061) and Africa/Middle East (8.4%; *n* = 1304).

Respiratory specimen sources were: sputum, 45.8% (*n* = 7088); endotracheal aspirate, 27.1% (*n* = 4190); bronchoalveolar lavage, 13.9% (*n* = 2146); bronchus, 8.2% (*n* = 1269). Less than 3% of isolates were classified as unspecified, thoracentesis fluid, lungs, trachea or aspirate.

The majority of isolates from respiratory specimens were collected from male patients (64.5%; *n* = 9972) and half were from patients aged 65 years and older (50.0%; *n* = 7723). The percentage of isolates collected from respiratory specimens on general wards (47.9%; *n* = 7398) was similar to intensive care units (43.4%; *n* = 6716).

### *Enterobacterales*

Antimicrobial activity and susceptibility data for a panel of antimicrobial agents against the collection of *Enterobacterales* isolates from respiratory sources are shown by region in Table 1. In each region, the highest rates of susceptibility among the collection of *Enterobacterales* were to ceftazidime-avibactam, meropenem, colistin and amikacin (≥94.4%). Tigecycline susceptibility in all regions was ≥97.9% among isolates of *C. koseri* and *E. coli*, the only species within the *Enterobacterales* collection to which EUCAST breakpoints apply.

**Table 1:** Antimicrobial activity against respiratory *Enterobacterales* isolates by region (ATLAS 2016–2018)

Region^**a**^	Antimicrobial	MIC (mg/L)		%S	%I	%R
		MIC_**90**_	Range			
Africa/Middle East (*n* = 833)	Amoxicillin-clavulanate	≥64	0.5–≥64	37.0	N/A	63.0
	Piperacillin-tazobactam	128	≤0.12–≥256	78.3	5.4	16.3
	Aztreonam	64	≤0.015–≥256	65.2	3.8	31.0
	Ceftazidime	64	≤0.015–≥256	65.5	4.8	29.7
	Ceftazidime-avibactam	0.5	≤0.015–≥256	99.0	N/A	1.0
	Cefepime	32	≤0.12–≥64	66.3	4.8	28.9
	Imipenem	2	0.06–≥16	89.9	8.0	2.0
	Meropenem	0.12	0.015–≥32	96.8	1.0	2.3
	Levofloxacin	≥16	0.03–≥16	71.9	8.6	19.4
	Colistin (*n* = 714)^a^	0.5	≤0.06–≥16	97.8	N/A	2.2
	Amikacin	4	≤0.25–≥128	95.8	N/A	4.2
	Tigecycline^b^	1	0.03–8	98.6	0.0	1.4
Asia/South Pacific (*n* = 2033)	Amoxicillin-clavulanate	≥64	≤0.12–≥64	47.2	N/A	52.8
	Piperacillin-tazobactam	128	≤0.12–≥256	76.7	4.7	18.5
	Aztreonam	128	≤0.015–≥256	66.9	2.9	30.2
	Ceftazidime	128	≤0.015–≥256	65.2	3.3	31.5
	Ceftazidime-avibactam	1	≤0.015–≥256	96.9	N/A	3.1
	Cefepime	32	≤0.12–≥64	74.0	3.3	22.7
	Imipenem	2	0.06–≥16	89.5	6.4	4.1
	Meropenem	0.12	0.015–≥32	95.5	1.0	3.4
	Levofloxacin	≥16	0.015–≥16	66.5	6.7	26.8
	Colistin (*n* = 1824)^a^	1	≤0.06–≥16	95.2	N/A	4.8
	Amikacin	4	≤0.25–≥128	94.9	N/A	5.1
	Tigecycline^b^	1	≤0.015–≥16	97.9	0.0	2.1
Europe (*n* = 6006)	Amoxicillin-clavulanate	≥64	0.25–≥64	41.1	N/A	58.9
	Piperacillin-tazobactam	128	≤0.12–≥256	75.5	4.7	19.8
	Aztreonam	128	≤0.015–≥256	71.3	2.6	26.1
	Ceftazidime	128	≤0.015–≥256	70.4	3.9	25.7
	Ceftazidime-avibactam	0.5	≤0.015–≥256	98.5	N/A	1.5
	Cefepime	32	≤0.12–≥64	76.0	3.8	20.1
	Imipenem	2	≤0.03–≥16	86.6	9.0	4.4
	Meropenem	0.12	0.008–≥32	95.0	1.3	3.7
	Levofloxacin	≥16	0.008–≥16	72.6	4.7	22.7
	Colistin (*n* = 5172)^a^	0.5	≤0.06–≥16	96.6	N/A	3.4
	Amikacin	8	≤0.25–≥128	94.4	N/A	5.6
	Tigecycline^b^	1	≤0.015–≥16	98.8	0.0	1.2
Latin America (*n* = 1256)	Amoxicillin-clavulanate	≥64	0.5–≥64	42.4	N/A	57.6
	Piperacillin-tazobactam	128	≤0.12–≥256	78.5	4.8	16.7
	Aztreonam	128	≤0.015–≥256	67.0	2.1	30.9
	Ceftazidime	64	≤0.015–≥256	66.4	3.7	29.9
	Ceftazidime-avibactam	0.5	≤0.015–≥256	99.6	N/A	0.4
	Cefepime	32	≤0.12–≥64	70.8	4.3	24.9
	Imipenem	2	0.06–≥16	88.4	6.8	4.8
	Meropenem	0.12	0.015–≥32	95.8	1.1	3.1
	Levofloxacin	≥16	0.015–≥16	68.5	5.7	25.8
	Colistin (*n* = 1074)^a^	0.5	≤0.06–≥16	96.6	N/A	3.4
	Amikacin	4	≤0.25–≥128	94.6	N/A	5.4
	Tigecycline^b^	1	0.06–8	100	0.0	0.0

*MIC* minimum inhibitory concentration; *MIC*_*90*_ MIC required to inhibit the growth of 90% of isolates; *%S* percent susceptible, standard dosing regimen; *%I* percent susceptible, increased exposure; *%R* percent resistant; *N/A* not applicable

^a^Isolates of *Morganella* spp., *Proteus* spp., *Providencia* spp. or *Serratia* spp. were excluded due to intrinsic resistance

^b^Tigecycline EUCAST breakpoints only apply to isolates of *Citrobacter koseri* and *Escherichia coli*

Among ESBL-positive *Enterobacterales*, susceptibility was highest to ceftazidime-avibactam and colistin (≥93.6%) **(**Table 2**)**. Susceptibility to tigecycline was high among ESBL-positive *C. koseri* and *E. coli* (98.8%). The susceptibility among ESBL/carbapenemase-positive *Enterobacterales* was highest to colistin and ceftazidime-avibactam (≥64.7%). In this subset, 92.9% of *C. koseri* and *E. coli* were susceptible to tigecycline. Among carbapenemase-positive/MBL-negative *Enterobacterales*, susceptibility was highest to ceftazidime-avibactam and colistin (≥74.1%). All carbapenemase-positive/MBL-negative *C. koseri* and *E. coli* were susceptible to tigecycline. For MBL-positive *Enterobacterales*, susceptibility was highest to colistin, and the susceptibility of MBL-positive *C. koseri* and *E. coli* to tigecycline was 90.0%. No isolates of MBL-positive *Enterobacterales* were susceptible to amoxicillin-clavulanate, ceftazidime, ceftazidime-avibactam or cefepime.

**Table 2:** Antimicrobial activity against resistant respiratory *Enterobacterales* isolates by subset (ATLAS 2016–2018)

Organism	Antimicrobial	MIC (mg/L)		%S	%I	%R
		MIC_**90**_	Range			
*Enterobacterales*, ESBL-positive (*n* = 1963)	Amoxicillin-clavulanate	≥64	1–≥64	14.5	N/A	85.5
	Piperacillin-tazobactam	≥256	≤0.25–≥256	40.4	12.5	47.1
	Aztreonam	≥256	0.06–≥256	0.7	4.2	95.1
	Ceftazidime	≥256	0.25–≥256	2.6	8.2	89.2
	Ceftazidime-avibactam	2	≤0.015–≥256	93.6	N/A	6.4
	Cefepime	≥64	≤0.12–≥64	2.9	7.4	89.8
	Imipenem	≥16	≤0.03–≥16	82.7	4.0	13.3
	Meropenem	16	0.015–≥32	84.1	3.3	12.6
	Levofloxacin	≥16	≤0.03–≥16	18.3	12.0	69.7
	Colistin (*n* = 1931)^a^	1	≤0.06–≥16	94.6	N/A	5.4
	Amikacin	32	≤0.25–≥128	82.5	N/A	17.5
	Tigecycline^b^	2	≤0.015–≥16	98.8	0.0	1.2
*Enterobacterales*, ESBL/carbapenemase- positive (*n* = 351)	Amoxicillin-clavulanate	≥64	1–≥64	0.6	N/A	99.4
	Piperacillin-tazobactam	≥256	0.25–≥256	1.7	0.3	98.0
	Aztreonam	≥256	0.12–≥256	1.1	2.0	96.9
	Ceftazidime	≥256	0.5–≥256	1.4	1.7	96.9
	Ceftazidime-avibactam	≥256	0.03–≥256	64.7	N/A	35.3
	Cefepime	≥64	≤0.12–≥64	1.1	2.6	96.3
	Imipenem	≥16	0.25–≥16	16.2	12.0	71.8
	Meropenem	≥32	0.03–≥32	18.8	13.7	67.5
	Levofloxacin	≥16	0.06–≥16	6.0	4.8	89.2
	Colistin (*n* = 346)^a^	≥16	≤0.06–≥16	79.2	N/A	20.8
	Amikacin	≥128	0.5–≥128	58.1	N/A	41.9
	Tigecycline^b^	2	0.06–8	92.9	0.0	7.1
*Enterobacterales*, carbapenemase positive/ MBL-negative (*n* = 362)	Amoxicillin-clavulanate	≥64	1–≥64	0.6	N/A	99.4
	Piperacillin-tazobactam	≥256	0.25–≥256	1.1	0.6	98.3
	Aztreonam	≥256	0.06–≥256	7.5	1.1	91.4
	Ceftazidime	≥256	0.25–≥256	7.5	3.3	89.2
	Ceftazidime-avibactam	4	≤0.015–≥256	98.9	N/A	1.1
	Cefepime	≥64	≤0.12–≥64	6.1	5.8	88.1
	Imipenem	≥16	0.25–≥16	19.3	14.6	66.0
	Meropenem	≥32	0.03–≥32	21.8	19.3	58.8
	Levofloxacin	≥16	0.06–≥16	7.2	5.2	87.6
	Colistin (*n* = 359)^a^	≥16	≤0.06–≥16	74.1	N/A	25.9
	Amikacin	64	0.5–≥128	59.1	N/A	40.9
	Tigecycline^b^	2	0.06–8	100	0.0	0.0
*Enterobacterales*, MBL-positive (*n* = 157)^c^	Amoxicillin-clavulanate	≥64	32–≥64	0.0	N/A	100
	Piperacillin-tazobactam	≥256	2–≥256	3.8	0.0	96.2
	Aztreonam	≥256	≤0.015–≥256	10.8	3.8	85.4
	Ceftazidime	≥256	32–≥256	0.0	0.0	100
	Ceftazidime-avibactam	≥256	16–≥256	0.0	N/A	100
	Cefepime	≥64	4–≥64	0.0	1.3	98.7
	Imipenem	≥16	1–≥16	7.0	8.3	84.7
	Meropenem	≥32	0.25–≥32	10.8	15.9	73.2
	Levofloxacin	≥16	0.06–≥16	10.2	3.2	86.6
	Colistin (*n* = 146)^a^	4	≤0.06–≥16	89.0	N/A	11.0
	Amikacin	≥128	0.5–≥128	42.7	N/A	57.3
	Tigecycline^b^	2	0.12–8	90.0	0.0	10.0

*MIC* minimum inhibitory concentration; *MIC*_*90*_ MIC required to inhibit the growth of 90% of isolates; *%S* percent susceptible, standard dosing regimen; *%I* percent susceptible, increased exposure; *%R* percent resistant; *N/A* not applicable; *ESBL* extended-spectrum β-lactamase; *MBL* metallo-β-lactamase

^a^Isolates of *Morganella* spp., *Proteus* spp., *Providencia* spp. or *Serratia* spp. were excluded due to intrinsic resistance

^b^Tigecycline EUCAST breakpoints only apply to isolates of *Citrobacter koseri* and *Escherichia coli*

^c^Isolates in this subset are carbapenemase-positive and MBL-positive

**Fig. 1:**
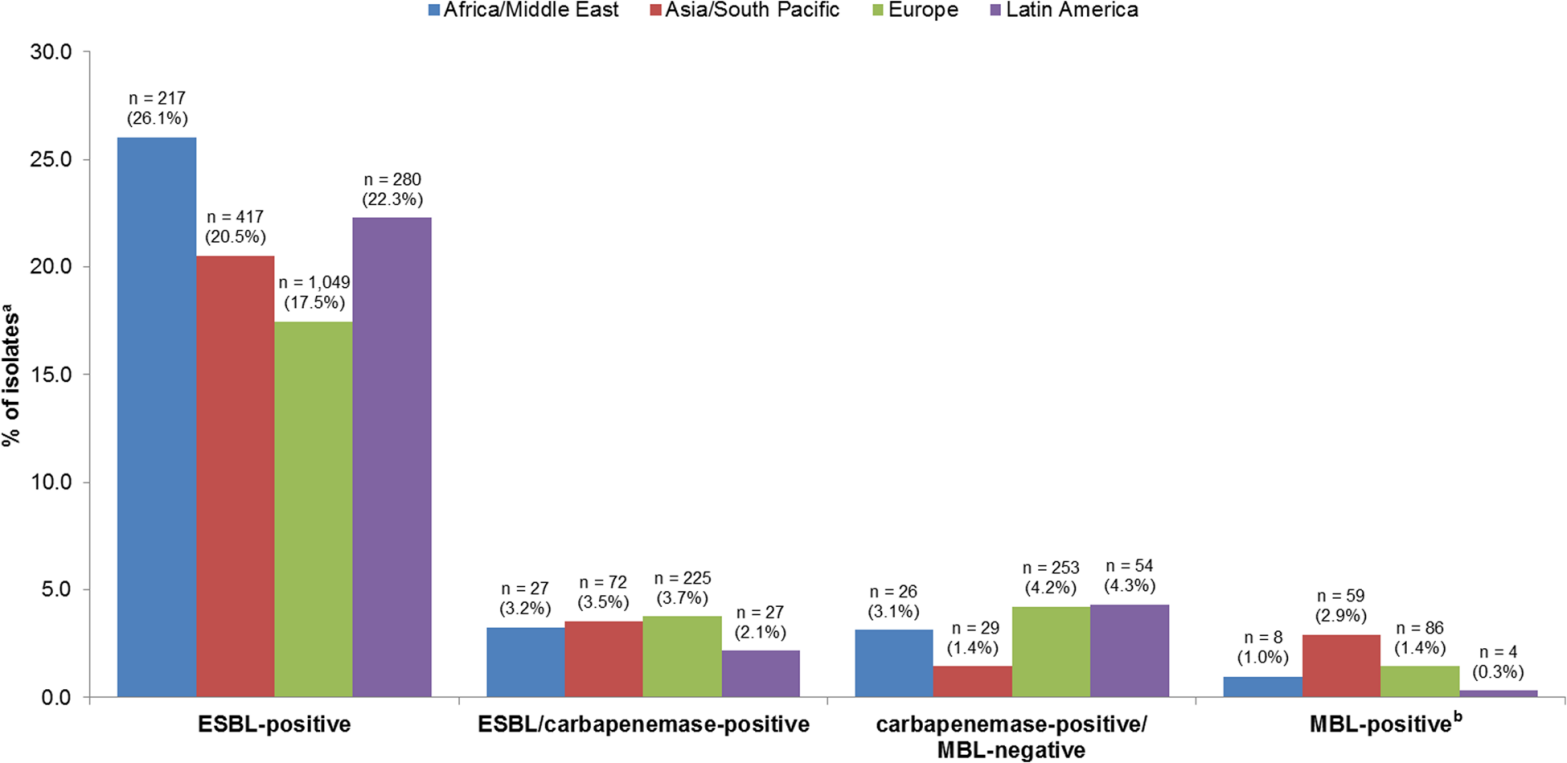
Rates of resistant respiratory *Enterobacterales* isolates by subset/region (ATLAS 2016–2018). ^a^Total isolate numbers (2016–2018) by region were: Africa/Middle East, 833; Asia/South Pacific, 2033; Europe, 6006; Latin America, 1256. ^b^Isolates in this subset are carbapenemase-positive and MBL-positive

By region, the 2016–2018 rate of ESBL-positive *Enterobacterales* was lowest in Europe and highest in Africa/Middle East **(**Fig. 1**)**. Fewer than 5% of *Enterobacterales* isolates collected in each region were ESBL/carbapenemase-positive, carbapenemase-positive/MBL-negative or MBL-positive.

### *Pseudomonas aeruginosa*

Antimicrobial activity and susceptibility data for a panel of antimicrobial agents against the collection of *P. aeruginosa* isolates from respiratory sources are shown by region in Table 3. The combined susceptibility (susceptible at standard dosing regimen plus susceptible at increased exposure) among the collection of *P. aeruginosa* in each region was highest to colistin, ceftazidime-avibactam and amikacin (≥82.4%). Rates of susceptibility to ceftazidime-avibactam, meropenem and amikacin were lower in Latin America than in Africa/Middle East, Asia/South Pacific and Europe.

**Table 3:** Antimicrobial activity against respiratory *Pseudomonas aeruginosa* isolates by region (ATLAS 2016–2018)

Region	Antimicrobial	MIC (mg/L)		%S + %I^**a**^	%R
		MIC_**90**_	Range		
Africa/Middle East (*n* = 471)	Piperacillin-tazobactam	128	0.25–≥256	73.5	26.5
	Aztreonam	32	0.06–≥256	79.8	20.2
	Ceftazidime	64	0.12–≥256	79.0	21.0
	Ceftazidime-avibactam	8	≤0.015–≥256	91.9	8.1
	Cefepime	32	≤0.12–≥64	76.6	23.4
	Imipenem	≥16	0.06–≥16	66.7	33.3
	Meropenem^b^	16	0.015–≥32	82.8	17.2
	Levofloxacin	≥16	0.03–≥16	65.2	34.8
	Colistin	2	0.12–4	99.8	0.2
	Amikacin	16	≤0.25–≥128	92.1	7.9
Asia/South Pacific (*n* = 1007)	Piperacillin-tazobactam	128	≤0.12–≥256	72.7	27.3
	Aztreonam	32	≤0.015–≥256	79.5	20.5
	Ceftazidime	64	0.06–≥256	76.3	23.7
	Ceftazidime-avibactam	8	≤0.015–≥256	93.4	6.6
	Cefepime	32	≤0.12–≥64	79.3	20.7
	Imipenem	≥16	0.12–≥16	80.1	19.9
	Meropenem^b^	8	0.008–≥32	90.1	9.9
	Levofloxacin	≥16	0.008–≥16	66.7	33.3
	Colistin	2	≤0.06–≥16	99.2	0.8
	Amikacin	8	≤0.25–≥128	95.6	4.4
Europe (*n* = 3049)	Piperacillin-tazobactam	128	≤0.12–≥256	69.2	30.8
	Aztreonam	32	0.03–≥256	76.5	23.5
	Ceftazidime	64	0.06–≥256	74.0	26.0
	Ceftazidime-avibactam	8	≤0.015–≥256	91.0	9.0
	Cefepime	32	≤0.12–≥64	76.0	24.0
	Imipenem	≥16	≤0.03–≥16	69.3	30.7
	Meropenem^b^	16	0.008–≥32	83.1	16.9
	Levofloxacin	≥16	0.015–≥16	59.9	40.1
	Colistin	1	≤0.06–≥16	99.7	0.3
	Amikacin	32	≤0.25–≥128	89.8	10.2
Latin America (*n* = 805)	Piperacillin-tazobactam	128	0.25–≥256	69.3	30.7
	Aztreonam	64	0.06–≥256	75.9	24.1
	Ceftazidime	128	0.06–≥256	71.2	28.8
	Ceftazidime-avibactam	32	0.03–≥256	85.7	14.3
	Cefepime	32	≤0.12–≥64	72.3	27.7
	Imipenem	≥16	0.12–≥16	62.6	37.4
	Meropenem^b^	16	≤0.004–≥32	76.6	23.4
	Levofloxacin	≥16	0.015–≥16	60.7	39.3
	Colistin	1	≤0.06–≥16	99.5	0.5
	Amikacin	64	≤0.25–≥128	82.4	17.6

*MIC* minimum inhibitory concentration; *MIC*_*90*_ MIC required to inhibit the growth of 90% of isolates; *%S* percent susceptible, standard dosing regimen; *%I* percent susceptible, increased exposure; *%R* percent resistant

^a^Includes all isolates not in the resistant category for all antimicrobial agents

^b^The breakdown of meropenem %S and %I data is: Africa/Middle East, 67.5% (%S) and 15.3% (%I); Asia/South Pacific, 79.7% (%S) and 10.3% (%I); Europe, 69.5% (%S) and 13.6% (%I); Latin America, 63.0% (%S) and 13.7% (%I)

Among MDR isolates of *P. aeruginosa*, susceptibility was highest to colistin, ceftazidime-avibactam and amikacin (≥61.3%) **(**Table 4**)**. Susceptibility among carbapenemase-positive/MBL-negative *P. aeruginosa* was highest to colistin and ceftazidime-avibactam (≥71.7%). No isolates of carbapenemase-positive/MBL-negative *P. aeruginosa* were susceptible to imipenem. All MBL-positive *P. aeruginosa* isolates were susceptible to colistin, with ≤19.1% susceptible to amikacin or ceftazidime-avibactam.

**Table 4:** Antimicrobial activity against resistant respiratory *Pseudomonas aeruginosa* isolates by subset (ATLAS 2016–2018)

Organism	Antimicrobial	MIC (mg/L)		%S + %I^**a**^	%R
		MIC_**90**_	Range		
*P. aeruginosa*, MDR (*n* = 1281)	Piperacillin-tazobactam	≥256	≤0.25–≥256	8.7	91.3
	Aztreonam	128	0.12–≥256	36.5	63.5
	Ceftazidime	≥256	0.06–≥256	19.1	80.9
	Ceftazidime-avibactam	64	≤0.015–≥256	62.9	37.1
	Cefepime	32	0.5–≥64	19.1	80.9
	Imipenem	≥16	≤0.03–≥16	17.1	82.9
	Meropenem	≥32	≤0.06–≥32	38.6	61.4
	Levofloxacin	≥16	≤0.03–≥16	9.6	90.4
	Colistin	1	≤0.06–≥16	99.5	0.5
	Amikacin	64	≤0.25–≥128	61.3	38.7
*P. aeruginosa*, carbapenemase positive/ MBL-negative (*n* = 53)	Piperacillin-tazobactam	≥256	16–≥256	3.8	96.2
	Aztreonam	≥256	2–≥256	28.3	71.7
	Ceftazidime	≥256	4–≥256	5.7	94.3
	Ceftazidime-avibactam	32	1–≥256	71.7	28.3
	Cefepime	≥64	4–≥64	15.1	84.9
	Imipenem	≥16	8–≥16	0.0	100
	Meropenem	≥32	8–≥32	3.8	96.2
	Levofloxacin	≥16	0.5–≥16	5.7	94.3
	Colistin	1	0.25–4	98.1	1.9
	Amikacin	64	2–≥128	30.2	69.8
*P. aeruginosa*, MBL-positive (*n* = 251)^b^	Piperacillin-tazobactam	≥256	4–≥256	5.6	94.4
	Aztreonam	64	0.25–≥256	56.6	43.4
	Ceftazidime	≥256	8–≥256	1.6	98.4
	Ceftazidime-avibactam	≥256	2–≥256	2.8	97.2
	Cefepime	≥64	8–≥64	4.4	95.6
	Imipenem	≥16	4–≥16	0.8	99.2
	Meropenem	≥32	4–≥32	3.2	96.8
	Levofloxacin	≥16	0.5–≥16	1.6	98.4
	Colistin	1	0.25–2	100	0.0
	Amikacin	≥128	2–≥128	19.1	80.9

*MIC* minimum inhibitory concentration; *MIC*_*90*_ MIC required to inhibit the growth of 90% of isolates; *%S* percent susceptible, standard dosing regimen; *%I* percent susceptible, increased exposure; *%R* percent resistant; *MDR* multidrug-resistant; *MBL* metallo-β-lactamase

^a^Includes all isolates not in the resistant category for all antimicrobial agents

^b^Isolates in this subset are carbapenemase-positive and MBL-positive

**Fig. 2:**
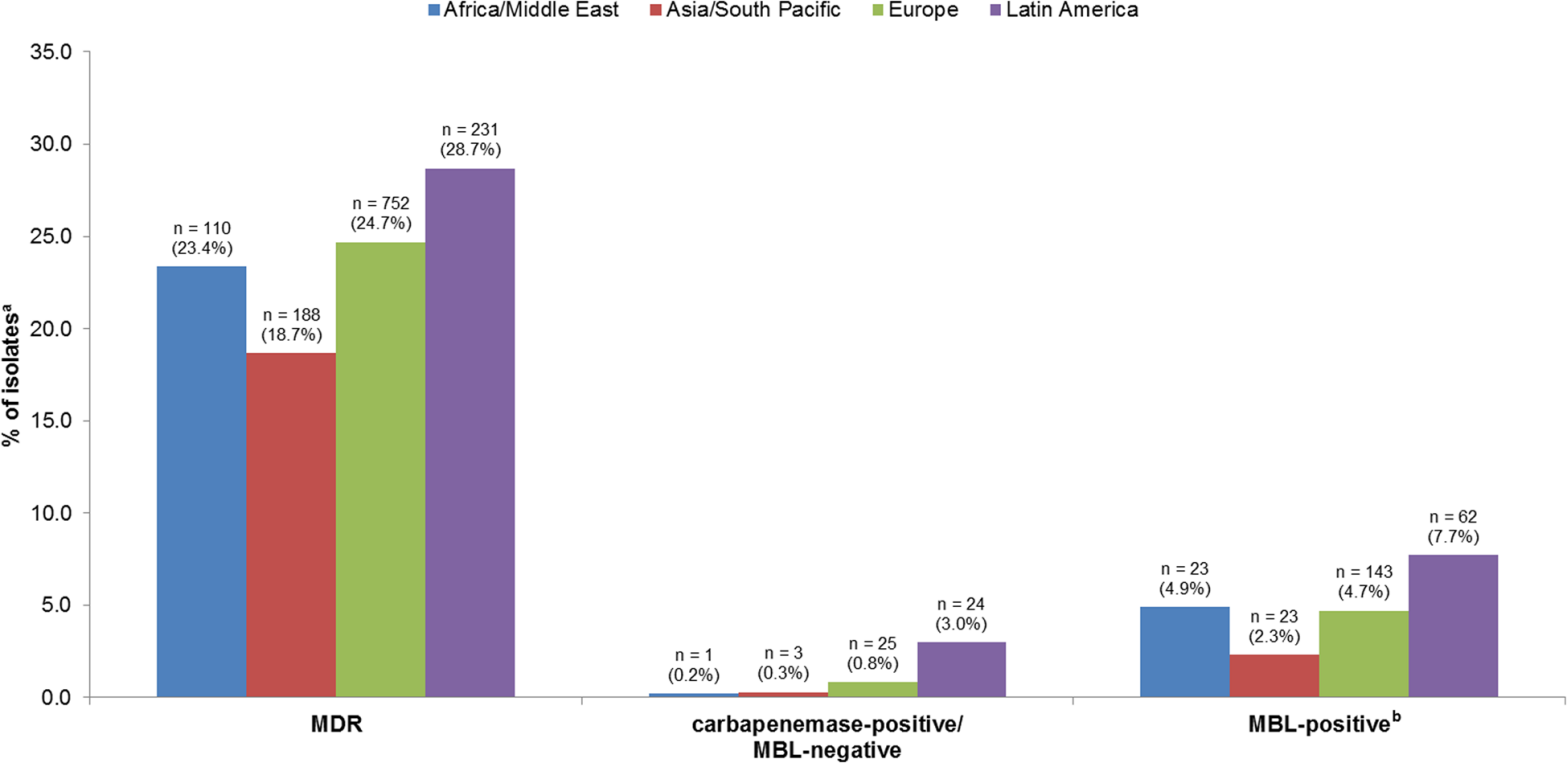
Rates of resistant respiratory *Pseudomonas aeruginosa* isolates by subset/region (ATLAS 2016–2018). ^a^Africa/Middle East, 471; Asia/South Pacific, 1007; Europe, 3049; Latin America, 805. ^b^Isolates in this subset are carbapenemase-positive and MBL-positive

By region, the 2016–2018 rate of MDR *P. aeruginosa* was lowest in Asia/South Pacific and highest in Latin America **(**Fig. 2**)**. The 2016–2018 rate of carbapenemase-positive/MBL-negative *P. aeruginosa* in each region was ≤3%, with only one such isolate collected in Africa/Middle East and three isolates in Asia/South Pacific. The 2016–2018 rate of MBL-positive *P. aeruginosa* was lowest in Asia/South Pacific and highest in Latin America.

## Discussion

This study presents in vitro antimicrobial activity and susceptibility data for a panel of antimicrobial agents against respiratory isolates of *Enterobacterales* and *P. aeruginosa*, collected as part of the ATLAS program (2016–2018), as well as data on subsets of resistant isolates.

Among the *Enterobacterales* isolates, rates of antimicrobial susceptibility to amikacin, ceftazidime-avibactam, colistin, meropenem and tigecycline were similar, irrespective of the geographical region of collection. For *P. aeruginosa* isolates, however, rates of susceptibility to ceftazidime-avibactam, colistin and amikacin were lower in Latin America, when compared with the other regions. In a phase 3 trial of hospitalized adults with HABP or VABP due to Gram-negative pathogens, the overall ceftazidime-avibactam MIC_90_ against isolates of *Enterobacterales* (*n* = 317) was 0.5 mg/L, and against isolates of *P. aeruginosa* (*n* = 101) was 8 mg/L [13]. These data are comparable with the MIC_90_ values for ceftazidime-avibactam in the current study, with the exception of isolates from Latin America, where the ceftazidime-avibactam MIC_90_ against the collection of *P. aeruginosa* was 32 mg/L.

The rates of all subsets of resistant *P. aeruginosa* isolates presented here were higher in Latin America than the other regions, which may explain the lower ceftazidime-avibactam activity and susceptibility seen in Latin America. The rate of MDR *P. aeruginosa* in Latin America (28.7%) was similar to a 2015–2016 global antimicrobial surveillance study (34.6%), where the rate in Latin America was the highest among seven geographical regions [14]. The 2015–2016 global study included aztreonam and colistin in their MDR study definition [14], whereas the current study definition of MDR *P. aeruginosa* omitted aztreonam and colistin, based on guidance from the Belgian High Council of Health [15] and the combinations of pathogens and antimicrobial agents under continued European Antimicrobial Resistance Surveillance Network (EARS-Net) surveillance [16]. A 2012–2015 study of clinical *P. aeruginosa* isolates from Latin America reported that 35.8% (643/1794) of *P. aeruginosa* isolates were meropenem-nonsusceptible (% intermediate plus % resistant) [17]; similar to the current study (37.0% [298/805]).

In the present study, susceptibility to ceftazidime-avibactam among carbapenemase-positive/MBL-negative *Enterobacterales* isolates remained high (98.9%). This was comparable to the 2012–2015 rate of ceftazidime-avibactam susceptibility reported among isolates of *Enterobacterales*, collected from the same regions as the current study, that were OXA-48-positive and MBL-negative (99.2%) [6]. The lack of ceftazidime-avibactam activity against MBL-positive isolates (due to the hydrolysis of both ceftazidime and avibactam by the MBL class of β-lactamases [18]) is clearly demonstrated in the present study. In addition, ceftazidime-avibactam susceptibility was notably lower among ESBL/carbapenemase-positive *Enterobacterales* (64.7%), compared with ESBL-positive isolates (93.6%). A total of 124 isolates in the ESBL/carbapenemase-positive subset were ceftazidime-avibactam-resistant, of which 123 were MBL-positive and the remaining isolate was carbapenemase-positive/MBL-negative (data not shown). The single carbapenemase-positive/MBL-negative isolate had a ceftazidime-avibactam MIC of 32 mg/L (EUCAST resistance breakpoint, > 8 mg/L). Isolates of *Enterobacterales* have been found to coproduce MBLs and Ambler class A β-lactamases, such as ESBLs [19].

The limitations of this study were the predefined number of isolates per species, as well as the variability in center and country participation between the study years, meaning that these results cannot be interpreted as epidemiology findings. The details on the type or size of study centers are not recorded at the time of isolate collection, which could limit the clinical relevance of the data. Despite this, the data reported here highlight the presence of antimicrobial-resistant respiratory Gram-negative pathogens in the four geographical regions presented. Ceftazidime-avibactam was active against resistant isolates of *Enterobacterales* and *P. aeruginosa*, with the exception of those organisms that were MBL-positive; among this subset of *Enterobacterales*, susceptibility was highest to colistin. This study provides valuable information to clinicians on the susceptibility of these resistant isolates to antimicrobial agents in current use. Continued monitoring of the antimicrobial susceptibility profiles of respiratory *Enterobacterales* and *P. aeruginosa* isolates is necessary to identify the most difficult-to-treat respiratory pathogens and improve patient outcomes.

## Conclusions

Among the collection of respiratory *Enterobacterales* isolates, rates of susceptibility to ceftazidime-avibactam, meropenem, colistin and amikacin were 94.4–99.6% in each region. For the subsets of resistant *Enterobacterales*, rates of susceptibility to ceftazidime-avibactam, meropenem and amikacin were lowest among MBL-positive isolates of *Enterobacterales* (0.0–42.7%), whereas colistin susceptibility was 89.0%. At least 90.0% of all *Citrobacter koseri* and *Escherichia coli* isolates were susceptible to tigecycline, including all subsets of resistant isolates.

For the collection of respiratory *P. aeruginosa* isolates, ceftazidime-avibactam, colistin and amikacin susceptibility was 82.4–99.8%. Among all resistant isolates of *P. aeruginosa*, colistin susceptibility remained ≥98.1%. For MBL-positive *P. aeruginosa*, antimicrobial susceptibility was lowest to all agents except colistin and aztreonam (0.0–19.1%).

For the majority of agents, antimicrobial susceptibility was reduced among the subsets of resistant Gram-negative isolates; most notably the MBL-positive subset. Monitoring of antimicrobial susceptibility is therefore necessary to help physicians to choose appropriate treatment options and improve the treatment outcomes for respiratory Gram-negative infections.

## Supplementary Information

**Additional file 1: Supplementary Table 1.**. Number of centers and respiratory isolates in participating counties (ATLAS 2016–2018). **Supplementary Table 2.** Species of respiratory *Enterobacterales* isolates (*n* = 10,128) (ATLAS 2016–2018).

## Data Availability

Data from the study can be accessed at https://atlas-surveillance.com. The datasets used and/or analyzed during the current study of isolates from respiratory specimens collected in Africa/Middle East, Asia/South Pacific, Europe and Latin America between 2016 and 2018, as part of the ATLAS program, are available from the corresponding author on reasonable request.

## Abbreviations

ATLAS: Antimicrobial Testing Leadership and Surveillance
ESBL: Extended-spectrum β-lactamase
EUCAST: European Committee on Antimicrobial Susceptibility Testing
HABP: Hospital-acquired bacterial pneumonia
I: Susceptible, increased exposure
KPC: *Klebsiella pneumoniae* carbapenemase
MBL: Metallo-β-lactamase
MDR: Multidrug-resistant
MIC: Minimum inhibitory concentration (mg/L)
MIC_50_: MIC required to inhibit growth of 50% of isolates (mg/L)
MIC_90_: MIC required to inhibit growth of 90% of isolates (mg/L)
N/A: Not available
PCR: Polymerase chain reaction
R: Resistant
S: Susceptible (standard dosing)
VABP: Ventilator-associated bacterial pneumonia
