# Supplementary material for: In vitro antimicrobial susceptibility of clinical respiratory isolates to ceftazidime-avibactam and comparators (2016–2018)
Source: BMC Infect Dis. 2021 Jun 23;21:600. doi: 10.1186/s12879-021-06153-0 (PMC8220879; doi:10.1186/s12879-021-06153-0)
Supplement: Supplementary file 1 — Additional file 1: Supplementary Table 1.. Number of centers and respiratory isolates in participating counties (ATLAS 2016–2018). Supplementary Table 2. Species of respiratory Enterobacterales isolates (n = 10,128) (ATLAS 2016–2018). [file 12879_2021_6153_MOESM1_ESM.docx]

Supplementary tables

Title:

In vitro antimicrobial susceptibility of clinical respiratory isolates to ceftazidime-avibactam and comparators (2016–2018)

List of authors:

Piérard D

Stone GG

# Supplementary Table 1. Number of centers and respiratory isolates in participating counties (ATLAS 2016–2018)

| **Region/Country** | **2016** | | **2017** | | **2018** | | **2016–2018** | | | |
| --- | --- | --- | --- | --- | --- | --- | --- | --- | --- | --- |
|  | **Center** | **Isolate** | **Center** | **Isolate** | **Center** | **Isolate** | **Center** | | **Isolate** | |
|  | **(n)** | **(n)** | **(n)** | **(n)** | **(n)** | **(n)** | **(n)** | | **(n)** | |
| **Africa/Middle East** | **10** | **371** | **11** | **435** | **19** | **498** | **22** | **1304** | |  |
| Israel | 3 | 154 | 4 | 164 | 4 | 169 | 5 | 487 | |  |
| Jordan | - | - | - | - | 1 | 14 | 1 | 14 | |  |
| Kuwait | 3 | 80 | 3 | 89 | 3 | 71 | 3 | 240 | |  |
| Morocco | - | - | - | - | 3 | 79 | 3 | 79 | |  |
| Nigeria | - | - | - | - | 3 | 11 | 3 | 11 | |  |
| Saudi Arabia | - | - | - | - | 1 | 18 | 1 | 18 | |  |
| South Africa | 4 | 137 | 4 | 182 | 4 | 136 | 6 | 455 | |  |
| **Asia/South Pacific** | **20** | **1072** | **21** | **951** | **34** | **1017** | **43** | **3040** | |  |
| Australia | 5 | 151 | 5 | 119 | 4 | 113 | 5 | 383 | |  |
| Hong Kong | - | - | - | - | 1 | 42 | 1 | 42 | |  |
| India | - | - | - | - | 8 | 158 | 8 | 158 | |  |
| Japan | 2 | 101 | 3 | 56 | 3 | 134 | 6 | 291 | |  |
| South Korea | 3 | 176 | 3 | 200 | 4 | 150 | 5 | 526 | |  |
| Malaysia | - | - | - | - | 3 | 51 | 3 | 51 | |  |
| Philippines | 4 | 234 | 4 | 277 | 3 | 110 | 6 | 621 | |  |
| Singapore | - | - | - | - | 1 | 5 | 1 | 5 | |  |
| Taiwan | 3 | 207 | 3 | 177 | 4 | 142 | 5 | 526 | |  |
| Thailand | 3 | 203 | 3 | 122 | 3 | 112 | 3 | 437 | |  |
| **Europe** | **67** | **2725** | **69** | **3098** | **104** | **3232** | **128** | **9055** | |  |
| Austria | 2 | 92 | - | - | - | - | 2 | 92 | |  |
| Belgium | 4 | 249 | 4 | 276 | 6 | 253 | 6 | 778 | |  |
| Croatia | - | - | - |  | 3 | 121 | 3 | 121 | |  |
| Czech Republic | 3 | 192 | 4 | 220 | 4 | 162 | 4 | 574 | |  |
| Denmark | 1 | 28 | 1 | 42 | 1 | 36 | 2 | 106 | |  |
| France | 8 | 341 | 7 | 310 | 10 | 299 | 12 | 950 | |  |
| Germany | 6 | 177 | 6 | 237 | 10 | 324 | 13 | 738 | |  |
| Greece | 3 | 76 | 3 | 124 | 3 | 54 | 4 | 254 | |  |
| Hungary | 3 | 129 | 3 | 171 | 4 | 149 | 5 | 449 | |  |
| Ireland | - |  | - | - | 3 | 95 | 3 | 95 | |  |
| Italy | 7 | 354 | 7 | 327 | 10 | 328 | 12 | 1009 | |  |
| Latvia | - | - | - | - | 1 | 33 | 1 | 33 | |  |
| Lithuania | - | - | - | - | 2 | 43 | 2 | 43 | |  |
| Netherlands | 2 | 67 | 2 | 83 | 2 | 48 | 3 | 198 | |  |
| Poland | 2 | 79 | 3 | 156 | 4 | 124 | 5 | 359 | |  |
| Portugal | 4 | 139 | 4 | 135 | 4 | 94 | 5 | 368 | |  |
| Romania | 1 | 55 | 1 | 6 | 4 | 49 | 4 | 110 | |  |
| Russia | 5 | 227 | 5 | 251 | 7 | 245 | 11 | 723 | |  |
| Spain | 6 | 218 | 8 | 315 | 11 | 359 | 12 | 892 | |  |
| Sweden | - | - | 1 | 56 | 1 | 38 | 1 | 94 | |  |
| Switzerland | - | - | - | - | 2 | 71 | 2 | 71 | |  |
| Turkey | 5 | 175 | 5 | 190 | 3 | 66 | 6 | 431 | |  |
| Ukraine | - | - | - | - | 2 | 69 | 2 | 69 | |  |
| United Kingdom | 5 | 127 | 5 | 199 | 7 | 172 | 8 | 498 | |  |
| **Latin America** | **22** | **705** | **23** | **661** | **30** | **695** | 41 | **2061** | |  |
| Argentina | 3 | 89 | 3 | 81 | 3 | 57 | 4 | 227 | |  |
| Brazil | 5 | 123 | 6 | 149 | 5 | 126 | 11 | 398 | |  |
| Chile | 3 | 102 | 2 | 106 | 3 | 75 | 3 | 283 | |  |
| Colombia | 2 | 40 | 3 | 70 | 5 | 100 | 6 | 210 | |  |
| Costa Rica | - | - | - | - | 1 | 11 | 1 | 11 | |  |
| Dominican Republic | - | - | - | - | 1 | 8 | 1 | 8 | |  |
| Guatemala | - | - | - | - | 2 | 57 | 2 | 57 | |  |
| Mexico | 6 | 238 | 6 | 171 | 6 | 167 | 7 | 576 | |  |
| Panama | - | - | - | - | 2 | 23 | 2 | 23 | |  |
| Venezuela | 3 | 113 | 3 | 84 | 2 | 71 | 4 | 268 | |  |
| **All** | **119** | **4873** | **124** | **5145** | **187** | **5442** | **234** | **15460** | |  |

# Supplementary Table 2. Species of respiratory *Enterobacterales* isolates (n = 10,128) (ATLAS 2016–2018)

| ***Enterobacterales* species** | **Isolates (n)** | **% of total** |
| --- | --- | --- |
| *Citrobacter amalonaticus* | 4 | 0.04 |
| *Citrobacter braakii* | 28 | 0.3 |
| *Citrobacter diversus* | 4 | 0.04 |
| *Citrobacter freundii* | 328 | 3.2 |
| *Citrobacter gillenii* | 1 | 0.01 |
| *Citrobacter koseri* | 262 | 2.6 |
| *Citrobacter*, non-speciated | 1 | 0.01 |
| *Enterobacter aerogenes* | 156 | 1.5 |
| *Enterobacter asburiae* | 132 | 1.3 |
| *Enterobacter cloacae* | 1064 | 10.5 |
| *Enterobacter hormaechi* | 1 | 0.01 |
| *Enterobacter kobei* | 52 | 0.5 |
| *Enterobacter ludwigii* | 10 | 0.1 |
| *Enterobacter*, non-speciated | 72 | 0.7 |
| *Escherichia coli* | 1766 | 17.4 |
| *Klebsiella aerogenes* | 406 | 4.0 |
| *Klebsiella oxytoca* | 602 | 5.9 |
| *Klebsiella pneumoniae* | 3667 | 36.2 |
| *Klebsiella variicola* | 193 | 1.9 |
| *Morganella morganii* | 173 | 1.7 |
| *Pantoea dispersa* | 1 | 0.01 |
| *Pluralibacter gergoviae* | 4 | 0.04 |
| *Proteus hauseri* | 22 | 0.2 |
| *Proteus mirabilis* | 288 | 2.8 |
| *Proteus penneri* | 2 | 0.02 |
| *Proteus vulgaris* | 107 | 1.1 |
| *Providencia alcalifaciens* | 1 | 0.01 |
| *Providencia rettgeri* | 44 | 0.4 |
| *Providencia stuartii* | 107 | 1.1 |
| *Raoultella ornithinolytica* | 26 | 0.3 |
| *Raoultella planticola* | 4 | 0.04 |
| *Serratia liquefaciens* | 4 | 0.04 |
| *Serratia marcescens* | 591 | 5.8 |
| *Serratia rubidaea* | 1 | 0.01 |
| *Serratia ureilytica* | 1 | 0.01 |
| *Serratia*, non-speciated | 3 | 0.03 |
